# Potential antitumor effect of cannabidiol (CBD) in canine oncology: a systematic review

**DOI:** 10.3389/fvets.2026.1800410

**Published:** 2026-05-21

**Authors:** Francisca J. Medina, Cristian G. Torres

**Affiliations:** 1 Centralized Laboratory for Veterinary Research (LACIV), Faculty of Veterinary and Animal Sciences, Universidad de Chile, Santiago, Chile; 2 Department of Clinical Sciences, Faculty of Veterinary and Animal Sciences, Universidad de Chile, Santiago, Chile; 3 Center of New Drugs for Hypertension and Heart Failure (CENDHY), División de Enfermedades Cardiovasculares, Pontificia Universidad Católica de Chile, Universidad de Chile, Universidad Andrés Bello, Santiago, Chile

**Keywords:** cancer, canine, cannabidiol, cannabinoid, dogs

## Abstract

**Introduction:**

Preparations of *Cannabis sativa* have been used for medicinal purposes for many centuries Currently, it is known that the phytocannabinoids present in the *Cannabis sativa* plant can modulate the endocannabinoid system, producing a variety of effects. Among the most abundant phytocannabinoids are delta-9-tetrahydrocannabinol (19-THC) and cannabidiol (CBD). CBD lacks psychotropic properties and has been shown to inhibit cell proliferation and migration, while inducing apoptosis in various human tumor cells. Studies evaluating CBD in dogs are more recent than those in humans, and to date, fewer publications are available. However, CBD has been shown to be safe and well-tolerated in dogs, supporting its potential clinical use. Since approximately 2015, some studies have been conducted evaluating CBD in different types of canine cancer; however, no comprehensive review of these findings has been performed.

**Methods:**

we conducted a systematic review Following the PRISMA 2020 guidelines.to compile the existing evidence on the anticancer effects of CBD in dogs.

**Results:**

We found that the studies conducted so far are pre-clinical, mostly based on cellular models, and that available data are primarily in lymphoma, mammary cancer, glioma, prostate cancer, osteosarcoma, and urothelial carcinoma. These studies consistently show that CBD exerts antiproliferative and proapoptotic effects, in some cases by modulating intracellular signaling pathways, including ERK, JNK, and caspases. Additionally, some studies have evaluated the combination of CBD with other drugs, reporting both synergistic and antagonistic effects. Overall, these findings highlight the potential of CBD as an anticancer agent across different cancer types.

**Discussion:**

Further studies are required to better elucidate the mechanisms underlying the effects of CBD and to standardize concentrations and formulations, enabling reliable, comparable results and the development of clinical studies evaluating the role of CBD in canine oncology.

## 1 Introduction

Preparations of *Cannabis sativa* have been used for many centuries for medicinal purposes; however, until the early 1990s, the mechanistic basis for cannabinoid action remained unknown. This changed significantly when specific cannabinoid receptors were successfully cloned, and their endogenous ligands were characterized, giving rise to what was termed the “Endocannabinoid System” (ECS) (1). This system is present in all vertebrates and is fundamentally composed of endogenous ligands, such as anandamide (AEA) and 2-arachidonoylglycerol (2-AG), enzymatic systems responsible for their synthesis and degradation, and cannabinoid receptors, forming a complex signaling system widely distributed throughout the organism. It is involved in multiple metabolic pathways and, in a versatile manner, regulates the physiology of cells in the central nervous system, the immune system, and other physiological systems (2, 69).

The ECS comprises a set of receptors, including cannabinoid receptor type 1 (CB1), cannabinoid receptor type 2 (CB2), G protein-coupled receptors 55 and 119 (GPR55, GPR119), transient receptor potential vanilloid (TRPV), and peroxisome proliferator-activated receptors (PPAR), forming what has recently begun to be referred to as the “endocannabinoidome” (3). The distribution of cannabinoid receptors is conserved across species: CB1 receptors are primarily found in the central and peripheral nervous systems, while CB2 receptors are mainly located in the immune system, although their distribution is not restricted to these tissues (4). In dogs, the presence of cannabinoid receptors or their ligands has been identified in embryos (5), the gastrointestinal tract (6, 7), the skin of healthy animals and those with atopic dermatitis (AD) (8, 9), the peripheral and central nervous systems (10, 11), and the joints (12). The localization of cannabinoid receptors in the brain has been associated with the physiological role of endocannabinoids in the control of movement and perception, the regulation of sleep and appetite, the inhibition of learning and memory processes, the regulation of emotional states, neuroprotection, and the potentiation of opioid action. Furthermore, the widespread distribution of the ECS allows it to participate in various homeostatic functions, exerting antioxidant, hypotensive, immunosuppressive, anti-inflammatory, and analgesic effects, as well as contributing to the regulation of vasomotor function, fertility, and tumor cell proliferation (13). Indeed, high expression of CB1 and CB2 receptors has been reported in low-grade cutaneous mast cell tumors, whereas high-grade cutaneous mast cell tumors in dogs show lower expression (14).

Phytocannabinoids, on the other hand, have been proposed as potential external regulators of the endocannabinoid system, capable in some cases of producing psychotropic effects, but also therapeutic ones. The most abundant phytocannabinoids are delta-9-tetrahydrocannabinol (Δ9-THC) and cannabidiol (CBD) (73). Currently, there is concern about the undesirable effects of Δ9-THC, the principal psychotropic component of the plant, including tachycardia, motor incoordination, somnolence, vomiting, and diarrhea, which have been reported in dogs as the main adverse effects associated with this molecule (15). CBD, on the other hand, lacks psychotropic activity and is a bicyclic, terpenophenolic compound of 21 carbons, formed after the decarboxylation of its precursor, cannabidiolic acid (CBDA). It exhibits affinity for a range of receptors, including CB1, CB2, GPR55, GPR119, TRPV, and PPAR, and demonstrates multiple therapeutic effects, including neuroprotective, antiepileptic, anxiolytic, antipsychotic, anti-inflammatory, analgesic, and anticancer properties (16, 61).

Regarding its clinical use in dogs, CBD has been explored in pathological conditions such as pain, particularly in osteoarthritis (17–19), seizures, neuroinflammation, and degenerative diseases (20), gastrointestinal disorders (21), anxiety-related conditions (74), dermatological diseases (75), immune disorders (76), and cancer (22–25). Additionally, safety and tolerability studies of CBD in dogs have shown that it is of low toxicity and produces mild or negligible adverse effects. To date, no oral therapeutic dose has been established, and its intravenous toxicity is low (LD50 >254 mg/kg) (26). Some studies evaluating oral CBD tolerability and safety in dogs, using doses ranging from 1 to 100 mg/kg, have reported few adverse effects, mainly associated with gastrointestinal discomfort, such as nausea, sialorrhea, decreased appetite, vomiting, and soft stools. This may be related to the oil-based vehicle used, as other formulations, such as liposomal preparations or CBD tablets, have not shown these signs (3). Additionally, the pharmacokinetics of doxorubicin have been studied in combination with CBD in canine patients diagnosed with high-grade lymphoma. In this context, the tolerability and safety of CBD were also evaluated during treatment administration, showing that the combination of CHOP chemotherapy protocol (cyclophosphamide, doxorubicin, vincristine, and prednisone) with CBD was well tolerated in the 19 dogs that completed the study, with no severe adverse events reported and no significant differences between groups in hematological and biochemical variables. Regarding doxorubicin pharmacokinetics, no significant variations were observed with CBD co-administration; however, the study evaluated only a single CHOP cycle (27). In general, most pharmacokinetic and safety studies have focused on assessing the safety of single-dose CBD or short- to medium-term administration (between 4 to 6 weeks) (28, 29, 62, 77), with only a few studies evaluating safety over periods of 3 months or longer (30, 31).

In the present study, we focused on reviewing oncological studies involving CBD in dogs, since the high incidence of this disease and the need for new therapeutic strategies (32). The annual incidence of cancer in dogs is estimated to be up to five times higher than in humans (1,000–2,500 vs. 500 new cases per 100,000, respectively), with more than 4 million new cancer diagnoses each year in the United States (32). Furthermore, it has been observed that dogs and humans share environments, nutrition, intact immune systems, cancer histology, therapeutic response, acquired resistance, recurrence, metastasis, and similar genetic and molecular targets (33). Therefore, the results observed with CBD in this pathology in dogs could help develop treatments in humans as well.

Regarding CBD in cancer, studies conducted primarily in human cells have demonstrated that micromolar doses (5–20 μM) of CBD can inhibit cell proliferation and migration while inducing apoptosis in various human tumor cells (34, 35). The antitumor effects described for CBD have been linked to its interaction with different membrane receptors, such as CB1, CB2, 5HT1A, GPR55, and TRPV1–4, as well as intracellular receptors such as PPARs and VDAC1 (35, 36, 67, 68, 78). Through these receptors, CBD can modulate various signaling pathways, including MAPK/ERK, mTOR-Akt, and COX-2-BCL2, among others (37, 63). However, the specific molecular mechanisms underlying the effect of CBD across different cancer types remain unclear, as does its therapeutic effectiveness as a single agent or in combination with other cannabinoids or chemotherapeutic drugs, as no standardized CBD-based pharmaceuticals have undergone clinical trials (38, 70).

The study of the antitumor properties of CBD in dogs is relatively recent, with studies available since 2019, compared with human studies conducted since the early 2000s. Consequently, there is currently a smaller body of evidence regarding the effects of CBD on canine cancer cells (22). The involvement of the endocannabinoid system has been studied only on canine mast cells, where increased expression of CB1 and CB2 receptors, or CB2 alone, has been reported in low-grade mast cell tumors (14, 79). Additionally, significantly higher levels of 2-AG have been identified in this type of cancer (80). The antitumor effect of CBD in dogs has been evaluated in cells from urothelial carcinoma, glioma, lymphoma, mammary carcinoma, prostate carcinoma, and osteosarcoma. These studies have assessed both isolated CBD and its use in combination with other cannabinoids or chemotherapeutic agents; however, no systematic review of these findings has been conducted. Therefore, in this systematic review, we aimed to examine the available evidence regarding the potential of CBD as an antitumor agent in dogs. To this end, we identified, compared, summarized, and developed a qualitative synthesis of the antineoplastic potential of CBD, with the objective of enhancing the current understanding and encouraging further research in this field.

## 2 Materials and methods

### 2.1 Study selection

A search for articles was conducted, encompassing experimental studies *in vitro* and *in vivo*, clinical trials, randomized controlled trials, and observational studies, such as case reports, case series, and bibliographic and systematic reviews, related to the therapeutic use of CBD in canine tumors.

### 2.2 Data collection techniques and instruments

Both authors (FJM and CGT) conducted a literature search across the PubMed, Web of Science, Scopus, Cochrane Library, BIOSIS Previews, and Embase databases in December 2025. The authors also conducted searches in Google Scholar and ProQuest. FJM repeated the search independently on January 18, 2026, to include any new studies. The authors collected studies and independently examined the titles, abstracts, and full texts obtained from the literature search, then compared and discussed the studies each identified as eligible for this review. The collected information was analyzed through a systematic review conducted in accordance with the PRISMA 2020 guidelines (64).

The data were organized in a comparative table that allowed contrasting therapeutic effects, doses used, routes of administration, reported adverse effects, and mechanisms of action explored in the use of CBD across the different studies. This analysis made it possible to establish patterns, similarities, and differences among the various studies regarding the therapeutic use of CBD applied to canine neoplasms.

### 2.3 Inclusion and exclusion criteria

The inclusion criteria for the selection of scientific literature were as follows:

- Scientific publications indexed in Q1, Q2, Q3, and Q4 journals.
- Studies conducted in dogs (*Canis lupus familiaris*) and in cancer cell lines from dogs
- Documents published between 2015 and 2025.
- Studies specifically addressing the use of CBD in canine tumors (studies conducted both in canine cancer cell lines and in dogs with cancer).

The exclusion criteria were:

- Non-scientific studies, from non-indexed journals, without a scientific committee.
- Studies that do not address the therapeutic use of CBD in canine neoplasms.

### 2.4 Data analysis

The collected information was analyzed to identify and categorize findings based on the central variables: cannabidiol, neoplasia, and canines. The systematic review was conducted according to the PRISMA (Preferred Reporting Items for Systematic Reviews and Meta-Analyses) 2020 guidelines.

A bibliographic record sheet was created to organize information from each source, including author, year, title, study type, relevant information, and conclusions. The data were organized into a comparative table that allowed the evaluation of therapeutic effects, doses used, routes of administration, reported adverse effects, and mechanisms of action explored in the use of CBD across the different studies. This analysis enabled the identification of patterns, similarities, and differences among the studies regarding the therapeutic use of CBD in canine tumors.

## 3 Results

**Figure 1:**
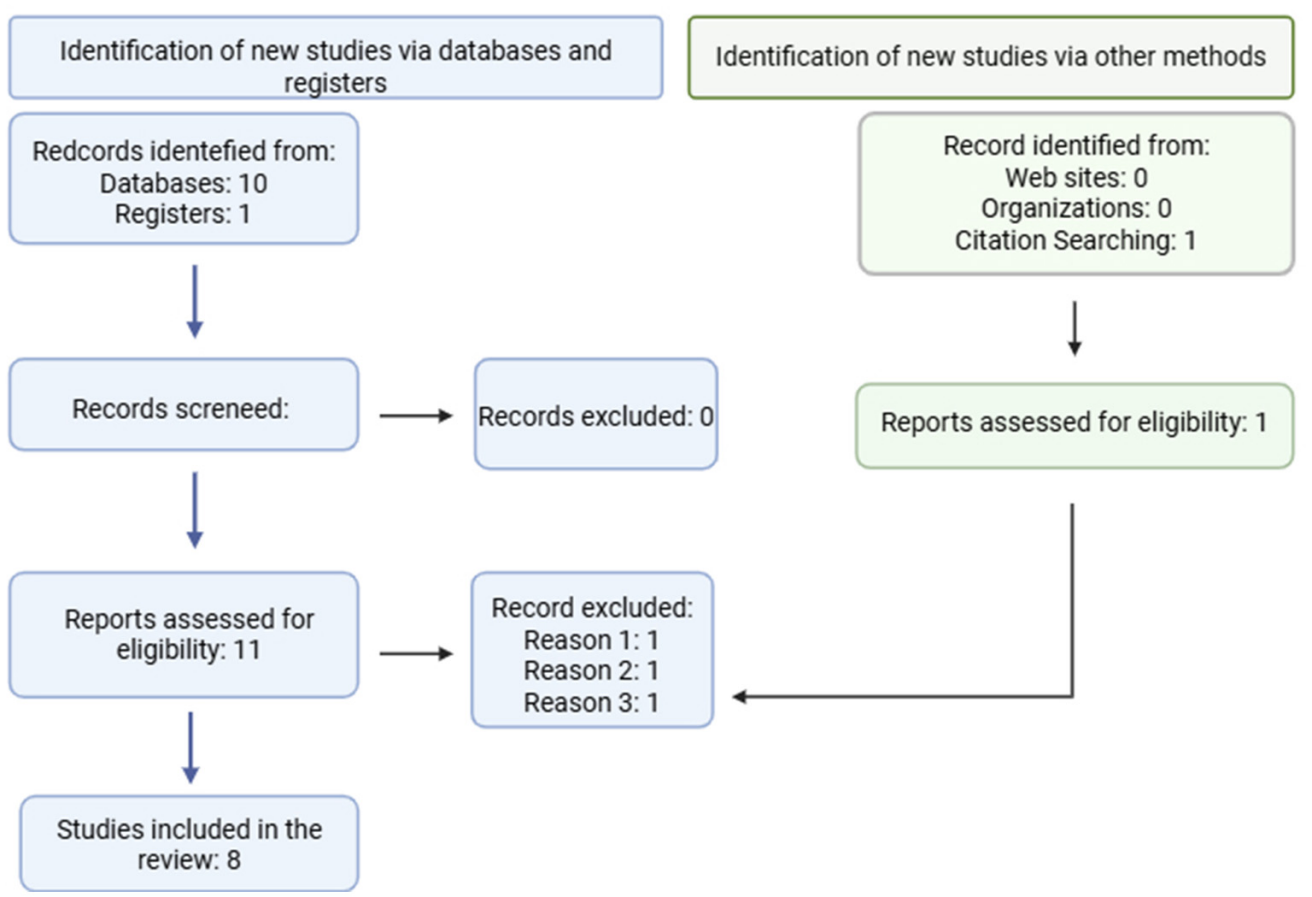
Flow diagram of the selection of scientific studies. Adapted from the PRISMA 2020 model (64).

As shown in Figure 1, the search across the selected databases yielded 11 articles associated with the defined keywords, of which 3 were excluded. One article was excluded because it reported a study using cannabinoids on canine cancer cells, but without CBD [Reason 1: (81)]. A second article was excluded because it was a review of the effects of cannabinoids on canine cancer but addressed their use as complementary palliative therapy without evaluating antitumor effects [Reason 2: (82)]. A third article was excluded because it was a clinical pharmacokinetic study of doxorubicin combined with CBD in dogs with lymphoma, where did not explore the anticancer potential of cannabinoids [Reason 3: (27)]. Therefore, this systematic review was conducted based on eight selected articles that met the search criteria.

The studies included in this review were all pre-clinical analyses evaluating the *in vitro* and *in vivo* antitumor effects of CBD on canine cancer cells of different anatomical origins. Table 1 summarizes these reports and briefly describes the main biological effects observed.

**Table 1:** Summary of studies showing *in vitro* and *in vivo* antitumor effects of CBD on different types of canine cancer cells.

Tipe of study	Type of cannabinoids used	Combination with other therapies	Concentration of CBD	Pharmacological effects	Receptors and molecular markers involved	References
*In vitro* (1,771 B-cell lymphoma cells cells)	CBD, CBDA and CBD-rich extract	Doxorubicin and vincristine	1.25–10 μg/ml	Reduction of viability and proliferation, induction of autophagy and apoptosis. Synergistic effects with vincristine	Increased activity ERK, JNK and caspases 3, 8 and 9	(22)
*In vitro* (1,771 and CLB-L1 B-cell lymphoma cells; CL-1 T-cell lymphoma cells)	CBD isolate, THC and WIN	—	10–25 μM	Reduction of viability, cytotoxicity, oxidative stress, and induction of apoptosis	Significant increase in nitrite content, reactive oxygen species (ROS), NADH, and H_2_O_2_, COX, increase caspase 8 and 9 activities; decrease in glutathione and complex-1	(24)
*In vitro* (1771 and CLB-L1 B-cell lymphoma cells)	CBD isolate, AEA and WIN	Cyclophosphamide, doxorubicin, vincristine and prednisone	0.1–50 μM	Reduction of viability. Synergistic effects of CBD at low doses and antagonistic effects at high doses	—	(72)
*In vitro* (CMT12 mammary carcinoma cells)	CBD, CBDA and CBD-rich extract	Doxorubicin and vincristine	1.25–10 μg/ml	Reduction of viability and proliferation, induction of autophagy and apoptosis. Synergistic effects with vincristine	Increased JNK phosphorylation, increased LC3II ratio, caspase 3 activation	(22)
*In vitro* (CF41.Mg and IPC366 mammary carcinoma cells)	CBD isolate and CBD nanoemulsion	—	20–50 μM	Reduction of viability and proliferation, cell cycle arrest in S-phase, increased apoptosis, and decreased migration and invasion	—	(25)
*In vitro* (HMPOS, D17, and Abrams osteosarcoma cells)	CBD, CBDA and CBD-rich extract	Doxorubicin and vincristine	1.25–10 μg/ml	Reduction of viability and proliferation, induction of autophagy and apoptosis. Synergistic effects with vincristine	Increased JNK phosphorylation, increased LC3II ratio, caspase 3 activation	(22)
*In vitro* (J3TBG and SDT3G glioma cells)	CBD isolate and CBD-rich extract	—	0.5–5 μg/ml	Reduction of viability, increased autophagic vesicles, cytotoxicity, and decreased mitochondrial function	Activation of the mitochondrial channel VDAC1, caspases, and RIPK3	(23)
*In vitro* (AXA, Orig and SH urotelial carcinoma cells)	CBD isolate	Mitoxantrone, vinblastine, carboplatin and piroxicam	5 μM	Reduction of viability, increased autophagic vesicles, cytotoxicity, and decreased mitochondrial function	—	(48)
*In vitro* (PC1 and PC2 prostate carcinoma cells)	CBD-rich extract and THC-rich extract	—	2.5–5 μM	Reduction of viability and increased apoptosis	—	(50)
*In vivo* (Mouse model, canine glioma cell J3TBG transplantation)	CBD isolate	Radiotherapy (RT)	30 mg/kg	Longer survival time in the CBD + RT group than in the RT group (not statistically significant)	—	(46)

### 3.1 Lymphoma

So far, three studies have evaluated the effects of CBD in lymphoma; all are *in vitro* studies that compare the effects of isolated CBD with other cannabinoids. Henry et al. (22) compared the effects of isolated CBD with CBDA and a CBD-rich extract containing lower concentrations of CBA, THC, and THCA on the 1717 cell line, whereas Omer et al. (72) compared isolated CBD with THC and a synthetic cannabinoid WIN 55-212-2 (WIN) on canine lymphoma cell lines (1,771, CLB-L1, and CL-1) and a human cell line (RAMOS). These studies differ in the formulation that achieves the greatest efficacy: Henry et al. reported greater reductions in cell viability with a CBD-rich extract, whereas Omer et al. (24) observed greater reductions with isolated CBD. However, despite both studies using the 1771 cell line, comparison of the results is difficult because different concentration units were used (Henry et al. used μg/ml, whereas Omer et al. used μM) (22, 24).

Henry et al. also evaluated the effect of isolated CBD in combination with chemotherapeutic agents, observing synergistic effects with vincristine at all tested doses (1.25, 2.5, 5, and 10 μg/ml), whereas with doxorubicin, synergistic effects were observed at high doses and antagonistic effects at low doses. Additionally, Omer et al. (72), using the 1771 cell line, analyzed the effects of isolated CBD, anandamide (AEA), and WIN both individually and in combination with CHOP protocol, observing, in contrast to Henry et al., synergistic effects of CBD at low doses and antagonistic effects at high doses (22, 72). These studies demonstrate differences in the effects of combining CBD with chemotherapeutic agents, suggesting that both beneficial and detrimental effects on treatment may occur and warrant further investigation.

At the mechanistic level, both Henry et al. (22) and Omer et al. (24) demonstrated pro-apoptotic effects, partly attributed to increased activity of caspases 8 and 9. Henry et al. also reported an increase in LC3II protein levels, indicative of autophagy induction. On the other hand, Omer et al. reported a significant increase in oxidative stress markers, including ROS, NADH, H_2_O_2_, and nitrite, following CBD treatment. Additionally, they observed increases in inflammatory markers (COX enzymes) and decreases in mitochondrial function markers (glutathione and complex I) (22, 24).

### 3.2 Mammary carcinoma

In mammary cancer, two *in vitro* studies have been conducted to date, using both isolated CBD, CBD combined with other cannabinoids (CBA, THC, and THCA), and CBD formulated in nanoemulsions. Overall, CBD has demonstrated the ability to reduce the viability of various canine mammary cancer cell lines, including CMT12 (derived from a Luminal B carcinoma), CF41.Mg (representative of triple-negative carcinoma), and IPC366 (an inflammatory carcinoma cell line). However, differences in the concentration units used (μg/ml vs. μM) and among the formulations (isolated CBD, CBD-rich plant extract, and nanoemulsion) complicate comparisons across studies (22, 25). Nevertheless, the effective doses observed in canine cells are similar to those reported in human and murine mammary cancer cells, which range between 5 and 20 μM (35). Medina et al. analyzed isolated CBD and CBD formulated in nanoemulsions on CF41.Mg and IPC366 cells, finding a more pronounced effect on cell viability with isolated CBD compared to the nanoemulsion, whereas Henry et al. reported better results in reducing the viability of the CMT12 cells with a CBD extract compared to isolated CBD. However, the nanoemulsion improved the interaction and internalization of CBD in CF41.Mg cells, demonstrating that it is an easily scalable and low-cost formulation (25, 39, 40).

An increase in apoptosis has also been observed with both isolated CBD and nanoemulsion formulations in CMT12 and CF41.Mg cells. In the study by Henry et al., increased caspase-3 activation and robust JNK phosphorylation, associated with apoptosis induction, were observed in CMT12 cells treated with isolated CBD. Meanwhile, Medina et al. reported S-phase cell cycle arrest and reduced cell migration with both isolated CBD and nanoemulsion formulations. These findings are consistent with results reported in human mammary cancer cells such as MDA-MB-231, where cell cycle arrest has been observed in G1, S–G2, and G2–M phases depending on the CBD concentration and exposure time (41, 42, 71, 83), as well as a reduction in migratory capacity in human cell lines 4T1.2, SUM159, and MDA-MB-231 treated with isolated CBD (34, 43).

### 3.3 Osteosarcoma

To our knowledge, only Henry et al. (22) have evaluated the effects of CBD on canine osteosarcoma *in vitro* using the HMPOS, D17, and Abrams cell lines. As in other cancer cell types, Henry et al. assessed the effects of CBD relative to CBDA and a CBD-rich extract containing lower concentrations of CBA, THC, and THCA. In this study, consistent with findings in canine mammary cancer and lymphoma cells, CBD in extract form was more effective in reducing the viability of the three osteosarcoma cell lines tested. Additionally, when the combination of isolated CBD with chemotherapeutic agents was analyzed, synergistic effects were observed with vincristine at all tested concentrations, whereas with doxorubicin, synergy was observed only at higher doses, with antagonistic effects at lower concentrations (22). In human osteosarcoma cell lines (MG-63 and U2R), synergistic effects between CBD and doxorubicin were observed, without evidence of antagonism (44). However, differences in the concentration units used (μg/ml vs. μM) complicate comparisons between results obtained in canine and human cells. Henry et al. also reported rapid induction of ERK and JNK phosphorylation in D17 cells, which was associated with apoptosis. Similarly, Li et al. found that CBD activated the PI3K-AKT-mTOR and MAPK pathways in human osteosarcoma cells (22, 44).

### 3.4 Glioma

In glioma, two studies have been published, one *in vitro* and one *in vivo*, involving the transplantation of canine glioma cells into mice. At the *in vitro* level, Gross et al. analyzed the effects of isolated CBD and a CBD-rich extract (containing 4% of other cannabinoids) in canine glioma cells J3TBG and SDT3G. In this study, the antiproliferative effects of CBD were evaluated, with isolated CBD showing greater reductions in cell viability than the CBD-rich extract. As part of the antiproliferative characterization, induction of apoptosis with isolated CBD was also observed (23). These findings are consistent with *in vitro* and *in vivo* studies conducted in human glioma cells, which have also demonstrated antiproliferative and pro-apoptotic effects (45). These antiproliferative and pro-apoptotic effects of CBD may be related to the increased survival time observed in mice in the *in vivo* study by Ukai et al., in which canine glioma cells (J3TBG) were transplanted into immunosuppressed mice treated with radiotherapy, isolated CBD, or a combination of both therapies. In these animals, higher CBD concentrations were observed in brain tissue than in plasma, and the group receiving combined CBD and radiotherapy showed increased survival time; however, no statistically significant differences were observed compared with radiotherapy alone (46).

Regarding the mechanisms underlying the observed effects of CBD, Gross et al. evaluated mitochondrial activity in the presence of isolated CBD and observed a reduction in oxygen consumption in J3TBG and SDT3G cells. Additionally, the cytosolic vesicles observed following CBD treatment were identified as swollen mitochondria, which are associated with the cytotoxic effects observed in these cells. This study also evaluated some pathways involved in cell death (apoptosis, necroptosis, and pyroptosis) using specific inhibitors, identifying the involvement of pan-caspases and RIPK3, a canonical necroptosis-associated kinase. However, the participation of caspase-1, a molecule implicated in pyroptosis, was not determined (23). Moreover, the involvement of the mitochondrial channel receptor VDAC1 and the TRPV1 receptor in apoptosis induction was assessed. Inhibition of VDAC1 restored cell viability in a manner similar to that observed following apoptosis inhibition, whereas no involvement of the TRPV1 receptor was identified (23). Studies in human glioma and glioblastoma cells have reported similar pro-apoptotic effects of CBD, as well as the involvement of the TRPV2 receptor (45, 47).

### 3.5 Urothelial carcinoma

In canine urothelial carcinoma, *in vitro* findings have been reported in a study that analyzed three cell lines (AXA, Orig, and SH), where the effects of CBD as monotherapy and in combination with chemotherapeutic agents (mitoxantrone, vinblastine, and carboplatin) or piroxicam were evaluated. In viability assays, CBD as monotherapy inhibited the viability of all three cell lines. Moreover, in combination therapies, CBD demonstrated synergistic effects with mitoxantrone and vinblastine across the three cell lines studied; however, the combination of CBD with carboplatin produced antagonistic effects at all tested concentrations in all cells evaluated. The combination of CBD with piroxicam did not significantly affect cell viability (48). This study also demonstrated increased apoptosis with the combinations of CBD plus vinblastine and CBD plus mitoxantrone; nevertheless, the potential molecular mechanisms underlying these effects were not studied. Anis et al. investigated the effects of CBD in human urothelial carcinoma cells, reporting a reduction in the viability of the T24 and HBT-9 cells, associated with an activation of CB1 and CB2 receptors (49).

### 3.6 Prostate carcinoma

In prostate carcinoma, *in vitro* evidence is currently limited to a single study that evaluated the effect of a CBD-rich extract containing small concentrations of other cannabinoids and unspecified terpenes, comparing its effect with a THC-rich extract. This study used the canine cell lines PC1 and PC2, in which a greater cytotoxic effect of the CBD-rich extract was observed compared to that of the THC-rich extract. It was also observed that CBD induced increased cell death; however, the type of cell death differed between the cell lines. Specifically, PC1 cells exhibited 36% cell lysis-related death, whereas in the PC2 line, the pre-dominant mechanism of cell death was late apoptosis (17.2%) (50). Studies in human prostate carcinoma using the LNCaP cell line have demonstrated primarily pro-apoptotic effects of CBD, associated with increased markers of intrinsic apoptotic pathways, including p53 upregulated modulator of apoptosis (PUMA), C/EBP homologous protein, intracellular Ca^2+^, p53, and reactive oxygen species (ROS) (De 50) (65, 66).

## 4 Discussion

According to the search criteria, eight studies evaluating CBD in canine tumors were selected (Figure 1), indicating that, so far, limited information is available in this field. Seven of these studies were conducted in cell lines, and only one was performed *in vivo* using a murine model (Table 1). Overall, the selected studies reported an antiproliferative effect of CBD, both *in vitro* and *in vivo*, highlighting its potential for treating different types of cancer in dogs. However, there is currently no consensus regarding the most appropriate formulation, as the reviewed studies have not evaluated standardized CBD formulations. Seven of the reviewed studies employed isolated CBD as part of their treatment protocols. Additionally, four studies (prostate carcinoma, lymphoma, osteosarcoma, and mammary carcinoma) evaluated alternative CBD formulations, including a nanoemulsion, full plant extracts rich in CBD, and other cannabinoids such as THC, CBDA, and WIN. Some studies reported improved outcomes with CBD extracts compared with isolated CBD (22, 50). CBD extracts contained varying proportions of other cannabinoids and terpenes; however, these studies did not clearly specify the concentration or composition of these compounds. This lack of detailed characterization limits the interpretation of results, as it remains difficult to determine the contribution of additional cannabinoids and terpenes to the observed effects. The use of full-spectrum plant extracts presents a challenge for translation into clinical studies involving CBD, as these extracts are difficult to reproduce consistently. Their composition varies depending on the cannabis plant type and cultivation conditions (51), making it challenging to replicate the exact cannabinoid and terpene profiles associated with favorable outcomes in canine tumors. Some studies in human breast cancer cells have also reported positive results using CBD-rich full-plant extracts, identifying β-caryophyllene as a terpene that may act synergistically with CBD to enhance its effects. However, comparisons between isolated CBD and extracts have yielded variable results, and differences in the composition of CBD extracts have further complicated the analysis of antitumoral responses (35, 36, 52, 53). The lack of standardization in formulation and the inconsistency in reported results have led to the current absence of approved CBD-based veterinary drugs. Regulatory agencies such as the FDA (United States) and the EMA (Europe) recommend the use of products for which comprehensive evidence regarding quality, safety, and efficacy is available (54).

Another challenge for the future translation of CBD into clinical studies in canine cancer lies in the concentrations to be administered. In general, pharmacokinetic studies in dogs have used oral doses ranging from 1 to 10 mg/kg, reporting plasma concentrations between 70 and 1,000 ng (0.223 nm−3.18 nm) (3, 55, 56). However, these plasma concentrations are substantially lower than those found to be effective in the studies we reviewed on CBD in canine cancer, indicating that, in general, higher doses would be required compared to those evaluated in safety studies or in efficacy studies for conditions such as epilepsy, pain, and behavioral disorders (3). Given that CBD has demonstrated a favorable safety profile to date, with pre-dominantly mild adverse effects reported in conducted trials, the implementation of clinical studies using higher doses may be feasible; however, this would increase the risk of adverse effects such as nausea, vomiting, diarrhea, and lethargy (3, 28, 29, 57). An additional limitation is the variability in concentrations used across studies. In *in vitro* experiments, most studies (5 of 8) used micromolar concentrations, reporting CBD IC50 values of approximately 2–10 μM. These concentrations are similar to those reported as effective against human cancer cells (53). The remaining studies used concentrations in the microgram range, with CBD IC50 values of approximately 2–10 μg/ml.

Regarding the use of CBD in combination with other drugs, three *in vitro* studies have evaluated its effects alongside chemotherapeutic agents. Inkol et al. assessed the effects of CBD combined with mitoxantrone, vinblastine, and carboplatin in urothelial carcinoma, observing synergistic effects with mitoxantrone and vinblastine, and antagonistic effects with carboplatin. On the other hand, Henry et al. evaluated the effect of CBD in combination with doxorubicin and vincristine, finding pre-dominantly synergistic effects with vincristine in lymphoma, osteosarcoma, and mammary carcinoma, and observing synergistic effects with doxorubicin at high CBD concentrations, but antagonistic effects at low concentrations. These results contrast with those reported by Omer et al. in 2025 in lymphoma, where, using the same cell line, they observed—contrary to Henry et al.—synergistic effects of CBD at low doses and antagonistic effects at high doses. These findings indicate that CBD does not induce uniform effects across different chemotherapeutic agents, as it may induce either synergistic or antagonistic interactions depending on concentration and cell type. However, because these studies used different cell lines and chemotherapeutic agents, direct comparisons cannot be made, and further studies are needed to confirm the reproducibility of these findings. This variability complicates the future development of clinical trials evaluating CBD in combination with chemotherapeutics, as the inconsistent results do not allow the establishment of a standardized CBD dose for use with drugs such as doxorubicin. Moreover, the antagonistic effects observed at low CBD doses would necessitate the use of higher doses, which may increase the risk of adverse effects, particularly gastrointestinal ones (28). In human cancer cells, CBD has been shown to sensitize cancer cells to cisplatin and significantly enhance cisplatin-mediated apoptosis, suggesting a potential adjuvant role for CBD (58). With respect to *in vivo* studies, to date, only Ukai et al. have evaluated the combination of CBD and radiotherapy in glioma, without identifying significant differences between treatments. In *in vivo* studies of human pancreatic cancer, CBD has been shown to enhance the effects of gemcitabine, with mice treated with the combination surviving nearly 3 times longer than those treated with gemcitabine alone (59).

An effect observed in most of the studies analyzed in this review is the induction of apoptosis. In five studies, cell death assays were performed, demonstrating that CBD significantly increases apoptosis across all evaluated cell lines. However, it is not possible to directly compare these results among studies, as different exposure times and CBD concentrations were used to assess this effect. In studies conducted in human cancer cells, including gastric, prostate, breast, lung, hepatocellular, ovarian, colorectal cancers, and gliomas, CBD has also been reported to induce apoptosis, although the doses and exposure times vary across studies (35, 36, 53).

Regarding the molecular mechanisms underlying the antitumor effects of CBD, limited information is currently available. Only three studies have evaluated signaling pathways associated with proliferation, autophagy, or apoptosis, and just one study has assessed the effects of CBD on the VDAC1 and TRPV1 receptors. In general, findings indicate effects related to mitochondrial stress (involving the VDAC1 receptor), increased phosphorylation of ERK and JNK associated with apoptosis induction, and increased LC3II levels, which are involved in autophagy induction. In prostate carcinoma, Calheiros et al. observed differences in the type of cell death induced by CBD between the PC1 cell line, described as non-metastatic, and the PC2 cell line, described as metastatic (50). Although the aforementioned study does not explain these differences, they may be attributable to the greater pharmacological sensitivity observed in the PC1 line and its dependence on receptor tyrosine kinases (RTKs) (PDGFR/VEGFR) (60). However, due to differences in the methodologies and endpoints evaluated across studies, direct comparison of results is not possible. Studies in various types of human cancers, including breast cancer, glioma, and prostate carcinoma, have further elucidated the molecular pathways affected by CBD. These studies show that CBD interacts with multiple cell surface and nuclear receptors (CB1, CB2, GPR55, TRPV1, and PPAR, among others), antagonizing the PI3K/AKT, MAPK/ERK, and JAK/STAT pathways. Additionally, CBD modulates calcium channels in cellular membranes and organelles, thereby altering intracellular signaling (45).

From this review, the authors identified a need for additional studies evaluating the effects of CBD in canine cancer, including *in vitro* studies to confirm the reproducibility of the results obtained to date, as well as investigations in other cancer types in which CBD has shown effects, such as lung and colorectal cancer. Furthermore, standardizing CBD concentrations and CBD extract formulations is necessary to enable comparisons across studies and facilitate the reliable translation of findings into clinical trials. Additionally, given that safety studies in dogs have shown that CBD is well-tolerated and presents a favorable safety profile, it is feasible to consider future clinical studies to evaluate its antitumor efficacy in dogs.

## 5 Conclusion

The findings from pre-clinical studies in dogs are consistent with those observed in humans, where CBD triggers antiproliferative and pro-apoptotic effects on several cancer cell types, which support clinical trials to elucidate the pharmacodynamics, pharmacokinetics, and potential antitumor efficacy of CBD in dogs with cancer.

## Data Availability

The original contributions presented in the study are included in the article/supplementary material, further inquiries can be directed to the corresponding author.
